# Prominent Literature in Surgical Ethics: A Comprehensive Bibliometric Analysis of the Top 50 Most-Cited Articles

**DOI:** 10.7759/cureus.87932

**Published:** 2025-07-14

**Authors:** Muhammad Hamza Shah, Sakshi Roy, Arjun Ahluwalia, Amer Harky

**Affiliations:** 1 Internal Medicine, Antrim Area Hospital, Antrim, GBR; 2 Medicine, School of Medicine, Dentistry and Biomedical Sciences, Queen's University Belfast, Belfast, GBR; 3 Cardiothoracic Surgery, Liverpool Heart and Chest Hospital, Liverpool, GBR

**Keywords:** bibliometric research, bioethics and ethics in research, medical humanities, operative ethics, surgical ethics

## Abstract

The patient-surgeon encounter is a complex relationship characterized by the infliction of pain for the patient's benefit. While surgical interventions are deeply personal, a significant knowledge disparity between patients and surgeons poses challenges in communication. Surgical training emphasizes the cultivation of virtues and practices to prioritize patient interests, but contemporary surgical practice still grapples with ethical quandaries. Addressing these challenges head-on is vital for advancing the field while ensuring patient safety, informed consent, cost-effectiveness, and conflict management. This article aims to contribute to the discourse on surgical ethics by delineating its scope, exploring key ethical issues, and examining strategies employed by surgeons. Additionally, it investigates the impact of surgical ethics on patients, surgeons, and society. Through a bibliometric analysis of the top 50 articles, key themes and influential works are identified. Themes include ethics of surgical innovation, history of surgical ethics, surgical ethics in practice, and ethics education. The analysis also reveals trends in countries of origin, authorship, article types, and journal representation. Findings inform the development of ethical frameworks, guidelines, and ethical competencies in surgical practice, promoting patient-centered care and professionalism. Collaboration among stakeholders is essential to shape the future of surgical ethics.

## Introduction and background

The patient-surgeon encounter is a distinctive and intricate relationship marked by the infliction of pain for the patient's benefit. Consequently, surgical interventions assume a deeply personal nature, intricately entwined with the idiosyncrasies of the individual surgeon. However, a significant disparity in knowledge exists between patients and surgeons, giving rise to subsequent challenges in communication. In order to prevent potential abuses and prioritize the best interests of patients, surgical training places great emphasis on the cultivation of virtues and practices [1]. Despite these conscientious efforts, contemporary surgical practice continues to face a number of ethical quandaries. Surgeons grapple with issues such as effectively communicating surgical uncertainty, nurturing patient-surgeon relationships, addressing ethical concerns in surgical training, and managing the impact of technology on end-of-life care [2].

In light of these considerations, the field of surgical ethics stands apart from other branches of medical ethics due to its unique characteristics and objectives. Ethical principles form an integral part of professionalism, as proficient surgeons are not only proficient in the technical aspects of surgery but also exhibit ethical reliability [3]. Ethical decision-making in the realm of surgery can be guided by approaches such as principlism and the four-box model, which offer structured frameworks for addressing ethical questions [4]. By tackling these ethical dilemmas head-on, the professional standing of surgeons is defined and the future of surgical progress is shaped. Upholding ethical standards allows surgeons to advance the field while ensuring patient safety, informed consent, cost-effectiveness, and the management of conflicts of interest [5].

This article endeavors to make a contribution to the discourse surrounding surgical ethics by stimulating thoughtful dialogue and inquiry. Its primary objectives are to delineate the scope of surgical ethics, explore key ethical issues encountered by surgeons, and examine the strategies employed to navigate these challenges. Moreover, it aims to investigate the ramifications of surgical ethics for patients, surgeons, and society as a whole. To facilitate this, a bibliometric analysis is utilized to quantitatively evaluate the published literature, providing insights into the trends, patterns, and impact of research within this specific field. By applying this methodology to the study of surgical ethics, researchers are able to systematically analyze the existing body of literature, identify central themes, and assess the scholarly contributions made in this area [6]. Such an approach enables a comprehensive understanding of the research landscape, shedding light on the most influential works, prolific authors, and emerging research directions. By scrutinizing citation patterns, publication trends, and co-authorship networks, researchers are able to gauge the influence and reach of specific articles and authors. This information aids in discerning which ethical issues have garnered the most attention, which concepts and approaches have gained widespread adoption, and which areas necessitate further exploration.

## Review

Methodology

Search Strategy and Selection Criteria

Between July 13, 2023, and July 15, 2023, searches were conducted in the SCOPUS database using the keyword "surgical ethics" to identify relevant articles published between 1985 and 2023. SCOPUS was selected as the database for this bibliometric analysis due to its extensive coverage of scholarly literature across various disciplines. The search results were then screened based on their titles and abstracts to identify articles directly related to surgical ethics. The screening process aimed to exclude articles that were not directly relevant to the research question. Two independent researchers, identified as SR and MHS, carefully examined the abstracts and/or full texts of the identified articles. The purpose of this evaluation was to determine if the articles investigated the topic of surgical ethics. Any disagreements between the two researchers regarding the inclusion or exclusion of an article were brought to the attention of a third researcher, identified as AA. The third researcher's role was to review the disputed cases and facilitate a consensus among the researchers.

Data Extraction

Parameters for each article were recorded, including the title of the article, year of publication, total number of authors, names of the authors (specifically, the first author and the corresponding author), number of citations, journal name, impact factor, country of authors, and types of articles. When authors hailed from different countries, the country of the corresponding author was chosen as the country of publication. This decision aimed to provide a standardized approach to categorizing the country associated with each article.

Data Analysis

To derive meaningful insights from the collected data, a comprehensive statistical analysis was conducted. This included descriptive statistics, keyword analysis, and qualitative analysis using established techniques to uncover patterns and relationships within the dataset. The statistical and qualitative procedures utilized in this study are detailed below, highlighting key aspects of the research methodology.

Descriptive statistics: Descriptive statistics, including mean ± SD for quantitative variables and median values (minimum-maximum) for a comprehensive understanding of the data's central tendency and variability, were employed. Categorical variables were presented as frequencies and percentages, providing an overview of their distribution in the dataset.

Keyword analysis: Keyword analysis was performed using VOSviewer software (version 1.6.19, Centre for Science and Technology Studies, Leiden University, The Netherlands), which facilitated the identification of key themes and patterns within the dataset. The software generated a visualization map based on the co-occurrence of keywords in the articles. 

Thematic content analysis: Qualitative analysis was conducted using the Braun and Clarke reflexive thematic analysis method [7]. This approach allowed for an in-depth exploration of the identified articles to identify recurring themes and patterns within the text. SR and AA engaged in a systematic process of familiarization with the data, followed by the generation of initial codes based on significant statements, ideas, or concepts related to surgical ethics. These initial codes were then organized into potential themes, which were refined and reviewed through iterative discussions among the researchers.

Results

**Figure 1:**
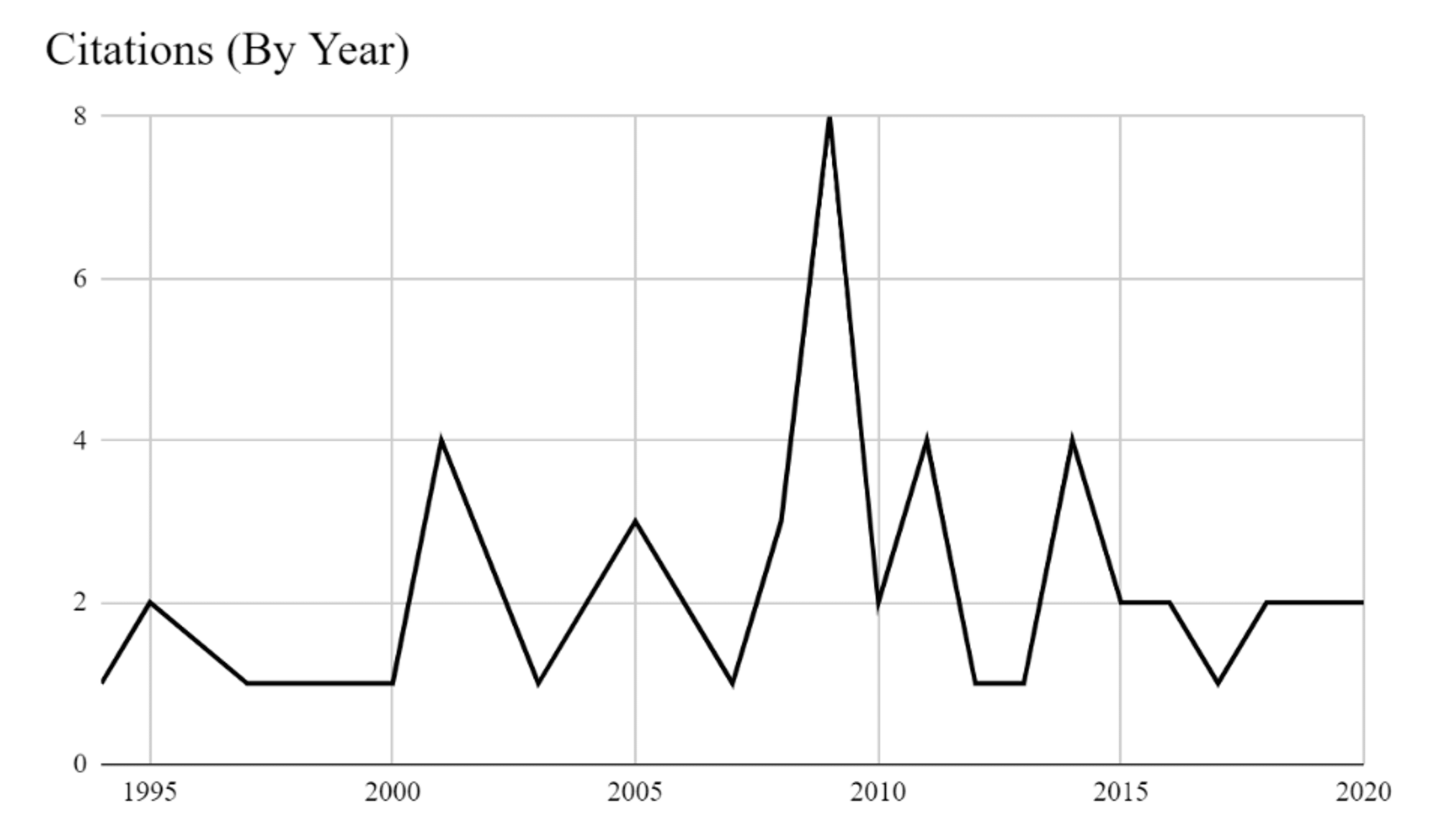
Citation count (with respect to the year of publication)

This comprehensive bibliometric analysis was conducted using the keyword "surgical ethics" in the SCOPUS database, covering a substantial time frame from 1953 to 2023. The search yielded a small pool of 183 articles relevant to the subject matter. From this small corpus, the top 50 most-cited articles were identified and are presented in Table 1. The average number of citations was determined to be 18.04±15.21 and the median number of citations was 14. Among these top 50 publications, the most-cited article was "Paediatric ethics and the surgical assignment of sex" by Kipnis and Diamond, published in 1998, with a total of 95 citations [8]. Furthermore, a detailed analysis revealed that the year 2009 witnessed the highest number of articles within the top 50 publications, comprising a total of nine articles. A graphical representation of the overall yearly breakdown can be found in Figure 1, providing a visual depiction of the publication trends over time.

**Table 1:** Top 50 most-cited articles (ranked in descending order) The "Affiliations" column represents the institutional affiliation(s) of the first author of each listed article.

Rank	Authors	Title	Year	Source Title	Citation Count	Affiliations	Document Type
1	Kipnis K, Diamond M [8]	Pediatric ethics and the surgical assignment of sex	1998	Journal of Clinical Ethics	95	Department of Philosophy, University of Hawaii, Manoa, HI, United States	Article
2	Jacobs JP, Cerfolio RJ, Sade RM [9]	The Ethics of Transparency: Publication of Cardiothoracic Surgical Outcomes in the Lay Press	2009	Annals of Thoracic Surgery	49	The Congenital Heart Institute of Florida, Division of Thoracic and Cardiovascular Surgery, All Children's Hospital, Saint Petersburg and Tampa, FL, United States	Article
3	Angelos P, DaRosa DA, Derossis AM, Kim B [10]	Medical ethics curriculum for surgical residents: Results of a pilot project	1999	Surgery	43	Department of Surgery, Northwestern University, Chicago, IL, United States;	Article
4	Ives J, Huxtable R [11]	Surgical ethics during a pandemic: moving into the unknown?	2020	British Journal of Surgery	39	Centre for Ethics in Medicine, Medical School, University of Bristol, United Kingdom	Note
5	Angelos P [12]	Ethics and surgical innovation: Challenges to the professionalism of surgeons	2013	International Journal of Surgery	36	MacLean Center for Clinical Medical Ethics, University of Chicago, United States	Review
6	Shiraz B, Shamim MS, Shamim MS, Ahmed A [13]	Medical ethics in surgical wards: knowledge, attitude and practice of surgical team members in Karachi.	2005	Indian Journal of Medical Ethics	33	Department of Surgery, Ziauddin Medical University Hospital, PECHS, Block 3, Karachi, 169-B, Pakistan	Article
7	Ramsey KM, Weijer C [14]	Ethics of surgical training in developing countries	2007	World Journal of Surgery	31	Cumberland Regional Health Care Centre, Amherst, NS, Canada	Article
8	Helft PR, Eckles RE, Torbeck L [15]	Ethics Education in Surgical Residency Programs: A Review of the Literature	2009	Journal of Surgical Education	28	Department of Medicine, Indiana University School of Medicine, Indianapolis, IN, United States	Review
9	Polgar S, Ng J [16]	Ethics, methodology and the use of placebo controls in surgical trials	2005	Brain Research Bulletin	27	School of Public Health, La Trobe University, Bundoora, Vic. 3038, Australia	Article
10	Angelos P [17]	Surgical ethics and the challenge of surgical innovation	2014	American Journal of Surgery	26	Department of Surgery and Surgical Ethics, University of Chicago Medicine, MC 4052, 5841 S. Maryland Avenue, Chicago, IL 60637, United States	Conference Paper
11	Jones JW [18]	Ethics of rapid surgical technological advancement	2000	Annals of Thoracic Surgery	24	Department of Surgery, University of Missouri, Columbia, MO, United States	Editorial
12	Thirunavukarasu P, Brewster LP, Pecora SM, Hall DE [19]	Educational intervention is effective in improving knowledge and confidence in surgical ethics-a prospective study	2010	American Journal of Surgery	22	Department of Surgery, University of Pittsburgh, Pittsburgh, PA, United States	Article
13	Angelos P [20]	Complications, errors, and surgical ethics	2009	World Journal of Surgery	21	Department of Surgery, MacLean Center for Clinical Medical Ethics, University of Chicago, 5841 S. Maryland Avenue, Chicago, IL 60637, United States	Review
14	Adedeji S, Sokol DK, Palser T, McKneally M [21]	Ethics of surgical complications	2009	World Journal of Surgery	21	Department of Primary Care and Social Medicine, Imperial College London, St. Dunstan's Road, London W6 8RP, United Kingdom	Article
15	Ashton CM, Wray NP, Jarman AF, Kolman JM, Wenner DM, Brody BA [22]	Ethics and methods in surgical trials	2009	Journal of Medical Ethics	20	Department of Surgery, The Methodist Hospital, Houston, Texas, United States	Article
16	Bendel O [23]	Surgical, therapeutic, nursing and sex robots in machine and information ethics	2015	Intelligent Systems, Control and Automation: Science and Engineering	19	School of Business, Institute for Information Systems, University of Applied Sciences and Arts Northwestern Switzerland FHNW, Basel, Switzerland	Article
17	Santiago C, Abdool S [24]	Conversations about challenging end-of-life cases: ethics debriefing in the medical surgical intensive care unit	2011	Dynamics (Pembroke, Ont.)	19	Critical Care Department, St. Michael's Hospital, Toronto, Ontario.	Article
18	Klingensmith ME [25]	Teaching Ethics in Surgical Training Programs Using a Case-Based Format	2008	Journal of Surgical Education	18	Department of Surgery, Washington University, St. Louis, MO, United States	Article
19	Namm JP, Siegler M, Brander C, Kim TY, Lowe C, Angelos P [26]	History and evolution of surgical ethics: John Gregory to the twenty-first century	2014	World Journal of Surgery	17	Department of Surgery, University of Chicago Medicine, 5841 S. Maryland Avenue, MC 6040, Chicago, IL, United States;	Article
20	Grossman E, Posner MC, Angelos P [27]	Ethics education in surgical residency: Past, present, and future	2010	Surgery	17	Department of Surgery, University of Chicago, Chicago, IL, United States	Article
21	Jones JW, McCullough LB, Richman BW [28]	Ethics of surgical innovation to treat rare diseases	2004	Journal of Vascular Surgery	17	Ctr. for Med. Ethics and Hlth. Plcy., Baylor College of Medicine, Houston, TX, United States	Article
22	Rahimi-Movaghar V, Saadat S, Vaccaro AR, Ghodsi SM, Samadian M, Sheykhmozaffari A, Safdari SM, Keshmirian B [29]	The efficacy of surgical decompression before 24 hours versus 24 to 72 hours in patients with spinal cord injury from T1 to L1 - With specific consideration on ethics: A randomized controlled trial	2009	Trials	16	Research Centre for Neural Repair, Sina Trauma and Surgery Research Center, Tehran University Medical Sciences, Tehran, Iran	Article
23	Gillett G [30]	Ethics of surgical innovation	2001	British Journal of Surgery	16	Otago Bioethics Centre, University of Otago Medical School, PO Box 913, Dunedin, New Zealand	Editorial
24	Little M [31]	Invited commentary: Is there a distinctively surgical ethics?	2001	Surgery	15	Centre for Values, Ethics and the Law In Medicine, University of Sydney, Australia	Article
25	Richardson DA [32]	Ethics in gynecologic surgical innovation	1994	American Journal of Obstetrics and Gynecology	15	Detroit, Michigan, United States	Article
26	Vercler CJ [1]	Surgical ethics: surgical virtue and more	2015	Narrative Inquiry in Bioethics	13	C. S. Mott Children's Hospital 1540 E Hospital Dr, Floor 3 Reception B, Ann Arbor, MI 48109, United States	Article
27	Larson JA, Johnson MH, Bhayani SB [33]	Application of surgical safety standards to robotic surgery: Five principles of ethics for nonmaleficence	2014	Journal of the American College of Surgeons	13	Division of Urologic Surgery, Washington University School of Medicine, 4960 Children's Pl, Campus Box 8242, St Louis, MO 63110, United States	Article
28	Howard F, McKneally MF, Upshur REG, Levin AV [34]	The formal and informal surgical ethics curriculum: Views of resident and staff surgeons in Toronto	2012	American Journal of Surgery	13	Joint Centre for Bioethics, University of Toronto, Toronto, ON, Canada	Article
29	Hassan AZ, Kadima KB, Remi-Adewumi BD, Awasum CA, Abubakar MT [35]	Animal models in surgical training: Choice and ethics	2005	Nigerian Journal of Surgical Research	12	Dept. of Veterinary Surgery, Dept. of Medicine, Veterinary Teaching Hospital Ahmadu Bello University, Zaria, Nigeria	Review
30	Paola F, Barten SS [36]	An 'ethics gap' in writing about bioethics: A quantitative comparison of the medical and the surgical literature	1995	Journal of Medical Ethics	12	Division of Internal Medicine, Nassau County Medical Center, East Meadow, NY, United States	Article
31	Angelos P [37]	The ethics of introducing new surgical technology into clinical practice the importance of the patient-surgeon relationship	2016	JAMA Surgery	11	Department of Surgery, MacLean Center for Clinical Medical Ethics, University of Chicago, 5841 S Maryland Ave, MC 4052, Chicago, IL 60637, United States	Short Survey
32	Bates T [38]	Ethics of consent to surgical treatment	2001	British Journal of Surgery	11	Breast Unit, William Harvey Hospital, Kennington Road, Willesborough, Ashford TN24 0LZ, United Kingdom	Short Survey
33	Williams JB, Mathews R, D'Amico TA [39]	"Reality surgery" a research ethics perspective on the live broadcast of surgical procedures	2011	Journal of Surgical Education	10	Division of Thoracic Surgery, Duke University Medical Center, DUMC 3496, Durham, NC 27710	Review
34	Little JM [40]	Ethics in surgical practice	2001	British Journal of Surgery	10	Centre for Values, Ethics and the Law in Medicine, University of Sydney, Sydney, NSW 2006, Australia	Short Survey
35	Kingham TP, Muyco A, Kushner A [41]	Surgical Elective in a Developing Country: Ethics and Utility	2009	Journal of Surgical Education	9	Memorial Sloan-Kettering Cancer Center, New York, NY, United States	Editorial
36	Jones JW, McCullough LB, Richman BW [42]	The ethics of innovative surgical approaches for well-established procedures	2004	Journal of Vascular Surgery	9	Ctr. for Hlth. Plcy. Med. Ethics, Baylor College of Medicine, Houston, TX, United States	Article
37	Leffall Jr LD [43]	Ethics in research and surgical practice	1997	American Journal of Surgery	9	Department of Surgery, Howard University Hospital, Washington, DC, United States	Conference Paper
38	Pollock RE, Curley SA, Lotzová E [44]	Ethics of research training for NIH t32 surgical investigators	1995	Journal of Surgical Research	9	Department of Surgical Oncology, University of Texas M.D. Anderson Cancer Center, 1515 Holcombe Boulevard, Houston, TX 77030, United States	Article
39	Karpowicz L, Bell E, Racine E [45]	Ethics oversight mechanisms for surgical innovation: A systematic and comparative review of arguments	2016	Journal of Empirical Research on Human Research Ethics	8	Institut de Recherches Cliniques de Montréal, Neuroethics Research Unit, 110 avenue des Pins Ouest, Montréal, QC H2WlR7	Review
40	Cardenas D [46]	Surgical ethics: A framework for surgeons, patients, and society	2020	Revista do Colegio Brasileiro de Cirurgioes	7	Faculty of Medicine, Universidad El Bosque, Research Institute in Nutrition, Genetics and Metabolism, Bogota, Colombia	Article
41	Teven CM, Grant SB [47]	Plastic surgery’s contributions to surgical ethics	2018	AMA Journal of Ethics	7	University of Chicago Medicine, United States	Article
42	Boult M, Fitzpatrick K, Maddern G, Fitridge R [48]	A guide to multi-centre ethics for surgical research in Australia and New Zealand	2011	ANZ Journal of Surgery	7	Department of Surgery, University of Adelaide, Woodville South, SA, Australia	Article
43	Angelos P [49]	Surgical ethics and the future of surgical practice	2018	Surgery (United States)	6	Department of Surgery and MacLean Center for Clinical Medical Ethics, The University of Chicago, Chicago, IL, United States	Article
44	Bagwell CE, Chiu P, Fecteau A, Gow KW, Mueller CM, Price D, Zigman AF [50]	2016 CAPS ethics session/Ein debate: 1. Regionalization of pediatric surgical care 2. Ethical introduction of surgical innovation 3. Addressing stress in a surgical practice: resiliency, well-being, and burnout	2017	Journal of Pediatric Surgery	6	Division of General and Thoracic Surgery, The Hospital for Sick Children, Toronto, ON, Canada	Review
45	Keune JD, Kodner IJ [51]	The importance of an ethics curriculum in surgical education	2014	World Journal of Surgery	6	Department of Surgery, Washington University School of Medicine, Campus Box 8109, 660 S. Euclid Ave., St. Louis, MO 63110, United States	Article
46	Brewster LP, Hall DE, Joehl RJ [52]	Assessing residents in surgical ethics: We do it a lot; We only know a little	2011	Journal of Surgical Research	6	Department of Surgery, Emory University Hospital, 1364 Clifton Road, Atlanta, GA 30022, United States	Article
47	Angelos P [53]	Orlo Clark and the rise of surgical ethics	2009	World Journal of Surgery	6	Department of Surgery, MacLean Center for Clinical Medical Ethics, University of Chicago, 5841 S. Maryland Avenue, Chicago, IL 60637, United States	Short Survey
48	Cosgrove DM [54]	Ethics in surgical innovation: vigorous discussion will foster future progress.	2008	Cleveland Clinic Journal of Medicine	6	Cleveland Clinic Foundation OH 44195, United States	Article
49	Ramsey KM [55]	International surgical electives: Reflections in ethics	2008	Archives of Surgery	6	Cumberland Eye Care, 4 Robert Angus Dr, Amherst, NS B4H 4R7, Canada	Note
50	Bernstein M, Bowman K [56]	Should a medical/surgical specialist with formal training in bioethics provide health care ethics consultation in his/her own area of speciality?	2003	HEC Forum	6	University of Toronto, Toronto Western Hospital, Toronto, Ontario, Canada M5T 2S8	Review

**Figure 2:**
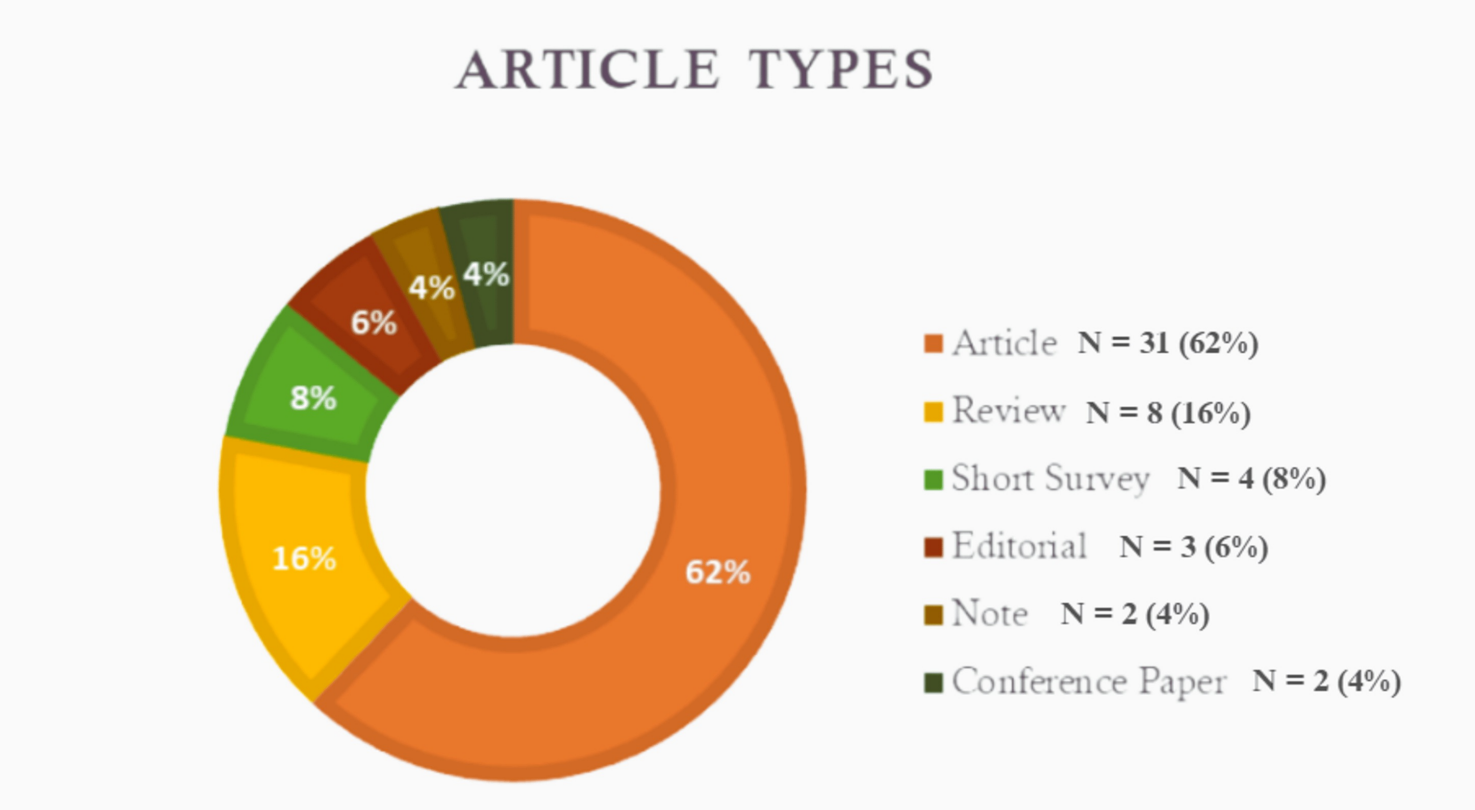
Article types (along with percentages)

Examination of article authorship revealed that Dr. Peter Angelos was a prolific contributor, with nine articles included in the Top 50 section along with 186 citations. Considering Angelos' significant standing on the list, his association with the Department of Surgery & Maclean Centre for Clinical Medical Ethics at the University of Chicago took precedence among the affiliations listed. Moreover, regarding the categorization of article types, original articles constituted the predominant category, encompassing 31 publications (Figure 2). When considering the countries of origin of the articles, the United States stood out with the highest number of contributions, amounting to 30 articles.

Upon analyzing the journals with the greatest proportion of articles among the top 50, it was observed that the World Journal of Surgery exhibited the highest representation, featuring six articles. Notably, the British Journal of Surgery boasted the highest impact factor among these journals. Furthermore, all the journals surveyed were classified within the Q1 category, with the exception of Brain Research Bulletin. A comprehensive breakdown of the article count, impact factor, and Q-indices for the top 10 journals can be found in Table 2.

**Table 2:** Journals in the top 50 most-cited list Values in the "Q-Index" column represent quartile rankings of journals based on their impact factor, as indexed in journal ranking databases such as Scimago Journal Rank (SJR) or Journal Citation Reports (JCR).

Journal Title	No. of Articles	Impact Factor	Q-Index
World Journal of Surgery	6	3.282	Q1
Journal of Surgical Education	4	2.900	Q1
British Journal of Surgery	4	5.572	Q1
American Journal of Surgery	4	2.403	Q1
Surgery	3	3.200	Q1
Journal of Surgical Research	2	2.200	Q1
Journal of Medical Ethics	2	2.021	Q1
Annals of Thoracic Surgery	2	5.113	Q1
Journal of Vascular Surgery	2	4.300	Q1
Brain Research Bulletin	1	3.751	Q2

**Figure 3:**
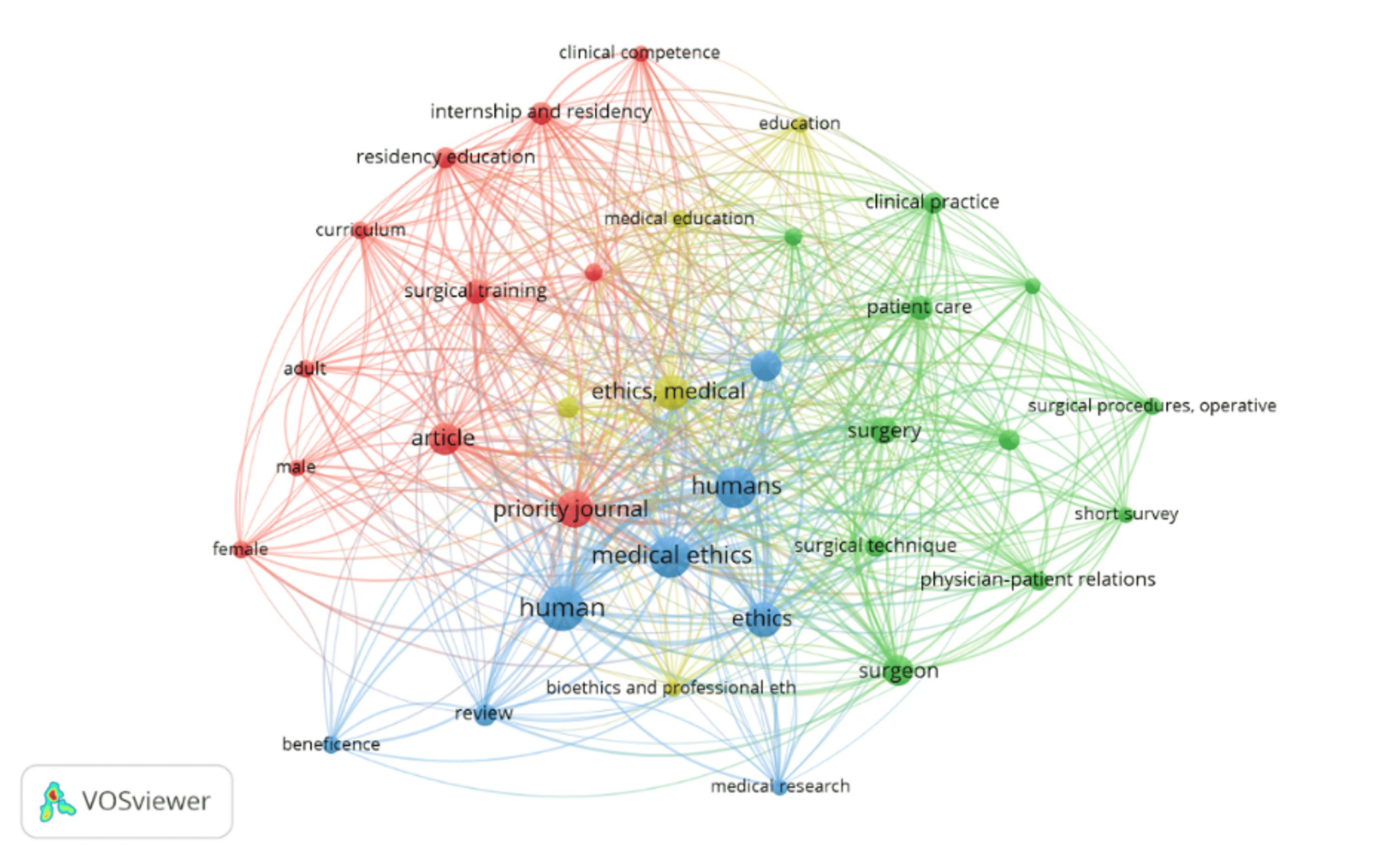
Keyword analysis and co-occurrence data Size of the nodes (bubbles): Represents the frequency of the corresponding keyword in the top-cited articles, larger bubbles indicate more frequent occurrence. Color of the nodes: Indicates different clusters of closely related keywords, based on co-occurrence patterns. Each color represents a group of terms that tend to appear together in the dataset. Thickness of the connecting lines: Denotes the strength or density of co-occurrence between two keywords, thicker lines mean a stronger relationship (i.e., those keywords appear together more often).

The 50 most frequently cited articles contained 35 primary keywords, as illustrated by the VOSviewer software. Among these, the most common ones were "Medical Ethics," "Human," "Humans," "Ethics," "Priority Journal," as well as "ethics, medical," and "surgical training" (Figure 3). In this visualization, the size of each bubble symbolizes the frequency of the corresponding keyword within these top-cited articles, while the connecting lines denote the density of co-occurrence with other highlighted keywords.

Finally, qualitative analysis, employing the Braun and Clarke reflexive thematic analysis method, unveiled four prominent themes within the field of surgical ethics. The first theme, ethics of surgical innovation, explored ethical considerations surrounding advancements in surgical techniques and technologies, including informed consent, patient safety, and conflicts of interest. The second theme focused on the history of surgical ethics, highlighting the evolution of ethical considerations in surgical practice and recognizing the contributions of influential medical ethicists, such as Thomas Percival. The third theme, surgical ethics in practice, examined ethical dimensions of the patient-surgeon relationship, encompassing informed consent, patient autonomy, and the ethical dilemmas encountered throughout the surgical journey. Finally, the fourth theme addressed ethics education in surgery, emphasizing the significance of comprehensive ethics curricula for surgical residents and surgeons in upholding professional and ethical standards.

Discussion

The present study conducted a bibliometric analysis to explore the landscape of surgical ethics literature, identify key themes and influential works, and examine trends and patterns within the field. The analysis involved a search on the SCOPUS database using the keyword "surgical ethics" and encompassed articles published between 1985 and 2023. A total of 183 relevant articles were identified, and the top 50 most-cited articles were analyzed in detail. In terms of article types, original articles constituted the majority, reflecting the emphasis on empirical research in the understanding of surgical ethics. It is also worth noting that original articles provide valuable insights into ethical issues faced by surgeons and contribute to the development of ethical frameworks and guidelines in surgical practice. In addition, our analysis also shed light on the countries of origin for these articles, with the United States leading in the number of contributions, accounting for 30 articles. This finding highlights a strong commitment to addressing ethical challenges in surgical practice and a robust research environment to go along with it. 

Furthermore, as outlined by the qualitative analysis, the ethics of surgical innovation was a recurring theme with the Top 50 articles. This involved ethical considerations surrounding the introduction of new surgical techniques, procedures, and technologies into clinical practice. As surgical progress relies on innovative solutions to patient problems, the introduction of new techniques and procedures raises ethical considerations [17,28]. The criteria for defining surgical progress have evolved beyond mere reductions in morbidity and mortality, as patients now value other factors such as improved quality of life and patient-centered outcomes. However, innovative procedures often come with uncertainties and unknown risks during the learning phase, leading to complex issues surrounding informed consent [30,45]. The cost-effectiveness of new techniques, which often depend on expensive technologies, also raises ethical concerns. Furthermore, collaborations between surgical device companies and surgeons can potentially introduce conflicts of interest that compromise patient well-being [12,30,32].

Not only this but two other important themes also emerged from the qualitative analysis: the history of surgical ethics and surgical ethics in surgical practice. Undoubtedly, the history of surgical ethics holds profound significance as it provides a window into the evolution of ethical considerations within surgical practice. As a result, understanding the historical context helps surgeons and researchers appreciate how ethical principles and practices have developed over time [57]. It provides insights into past ethical challenges, ethical frameworks, and landmark contributions to the field. Thomas Percival was often mentioned within this context and Percival's code of medical ethics, including its emphasis on surgical ethics, provided a foundation for subsequent developments in the field [58]. In his code, Percival addressed several ethical aspects relevant to surgical practice, such as the duty of surgeons to act in the best interest of their patients, the importance of honesty and truthfulness in communicating with patients, and the ethical responsibility to maintain patient confidentiality. Percival's work also touched upon issues of informed consent and the ethical considerations surrounding experimental surgeries and human subject research [59].

Conversely, the theme of surgical ethics in surgical practice focuses on the application of ethical principles and guidelines in the daily practice of surgery. This theme is important because it recognizes that ethical considerations extend beyond theoretical discussions and must be integrated into the practical realities of surgical care. Surgeons encounter numerous ethical challenges in their day-to-day work, such as patient-surgeon relationships, informed consent, decision-making in emergency situations, resource allocation, and end-of-life care. Understanding and addressing these ethical challenges is crucial for ensuring patient-centred care, maintaining professionalism, and upholding the trust between patients and surgeons [3,4]. Ethical reflection and decision-making in surgical practice require a comprehensive understanding of the ethical principles, values, and virtues that guide surgical care [60]. By exploring the theme of surgical ethics in surgical practice, surgeons can enhance their ethical competencies, promote patient welfare, and cultivate a culture of ethical practice within the surgical community [61].

The results of this analysis have implications for the advancement of surgical ethics and the professional standing of surgeons. Ethical considerations are essential in promoting patient safety, ensuring informed consent, addressing cost-effectiveness concerns, and managing conflicts of interest. By upholding ethical standards and engaging in thoughtful dialogue, surgeons can navigate the ethical challenges posed by surgical innovation, patient-surgeon relationships, communication of surgical uncertainty, and the impact of technology on end-of-life care. Consequently, identification of key themes and influential works in surgical ethics can guide future research and inform the development of ethical frameworks and guidelines in surgical practice. Further exploration of underrepresented topics and the examination of emerging ethical issues can contribute to the ongoing dialogue and foster the ethical development of surgical care.

Limitations

The paper focuses solely on the analysis of existing literature through bibliometric methods. While this approach offers valuable insights into trends and impact, it may not capture the entire landscape of ethical issues in surgical practice. Additionally, the analysis focuses on a specific time frame (1985-2023) and a single database (SCOPUS) for data collection. This may limit the generalizability of the findings to the broader field of surgical ethics. Including other databases and expanding the time frame could enhance the generalizability of the study.

## Conclusions

In conclusion, this study highlights the significance of surgical ethics in shaping the future of surgical practice. Ethical considerations are integral to patient-centered care, professionalism, and the advancement of surgical innovation. By upholding ethical standards and actively addressing ethical challenges, surgeons contribute to the ethical development of surgical care and ensure the highest level of patient care. For this purpose, collaboration among stakeholders is paramount in shaping the future of surgical ethics. Therefore, surgeons, ethicists, policymakers, patients, and industry representatives should engage in multidisciplinary discussions to develop guidelines and policies that promote ethical practice, transparency, and patient-centered care. 
